# *snHiC*: a complete and simplified snakemake pipeline for grouped Hi-C data analysis

**DOI:** 10.1093/bioadv/vbad080

**Published:** 2023-06-21

**Authors:** Sebastian Gregoricchio, Wilbert Zwart

**Affiliations:** Division of Oncogenomics, Netherlands Cancer Institute, Oncode Institute, 1066CX Amsterdam, The Netherlands; Division of Oncogenomics, Netherlands Cancer Institute, Oncode Institute, 1066CX Amsterdam, The Netherlands

## Abstract

**Summary:**

Genome-wide chromosome conformation capture (Hi-C) is a technique that allows the study of 3D genome organization. Despite being widely used, analysis of Hi-C data is technically challenging and involves several time-consuming steps that often require manual involvement making it error prone, potentially affecting data reproducibility. In order to facilitate and simplify these analyses we implemented *snHiC*, a snakemake-based pipeline that allows for the generation of contact matrices at multiple resolutions in one single run, aggregation of individual samples into user-specified groups, detection of domains, compartments, loops and stripes and performance of differential compartment and chromatin interaction analyses.

**Availability and implementation:**

Source code is freely available at https://github.com/sebastian-gregoricchio/snHiC. A yaml-formatted file (snHiC/workflow/envs/snHiC_conda_env_stable.yaml) is available to build a compatible conda environment.

**Supplementary information:**

Supplementary data are available at *Bioinformatics Advances* online.

## 1 Introduction

In the past decades, the epigenetics field has been revolutionized by the introduction of new techniques that allow for the interrogation of long-range DNA–DNA interactions and enable researchers to study the genome organization in three-dimensional space. Genome-wide chromosome conformation capture (Hi-C) (Lieberman-Aiden *et al.*, 2009) is considered one of the most advanced 3D genome analysis technologies. Hi-C, coupled to high-throughput sequencing, allows for the identification of DNA–DNA physical contacts that occur between regions contacting in 3D genome space while localized several megabase-pairs (Mb) away from each other in the linear genome.

The use of Hi-C in biomedical research is quickly increasing, but the analysis of these data remains challenging. Indeed, in contrast to RNA-seq and Chromatin ImmunoPrecipitation (ChIP)-seq data for which golden standard file formats are available, many different tools with their corresponding formats exist for the analysis of Hi-C data.

While many different pipelines are available for the analysis of Hi-C data [reviewed in Han and Wei (2017) and Hansen *et al.* (2019)], only few of them include a comprehensive generation of downstream data such as Topologically Associated Domains (TADs) calls, loops and stripes calls, A/B compartments and differential compartmentalization and interactions (Supplementary Table S1).

With the implementation of *snHiC* (publicly available at https://github.com/sebastian-gregoricchio/snHiC) we aim to fill these gaps by providing a user-friendly snakemake-based pipeline for a complete and customizable analysis of Hi-C data with the possibility to automatically repeat the same analyses by sample group and multiple resolutions.

## 2 Implementation

**Figure 1.**
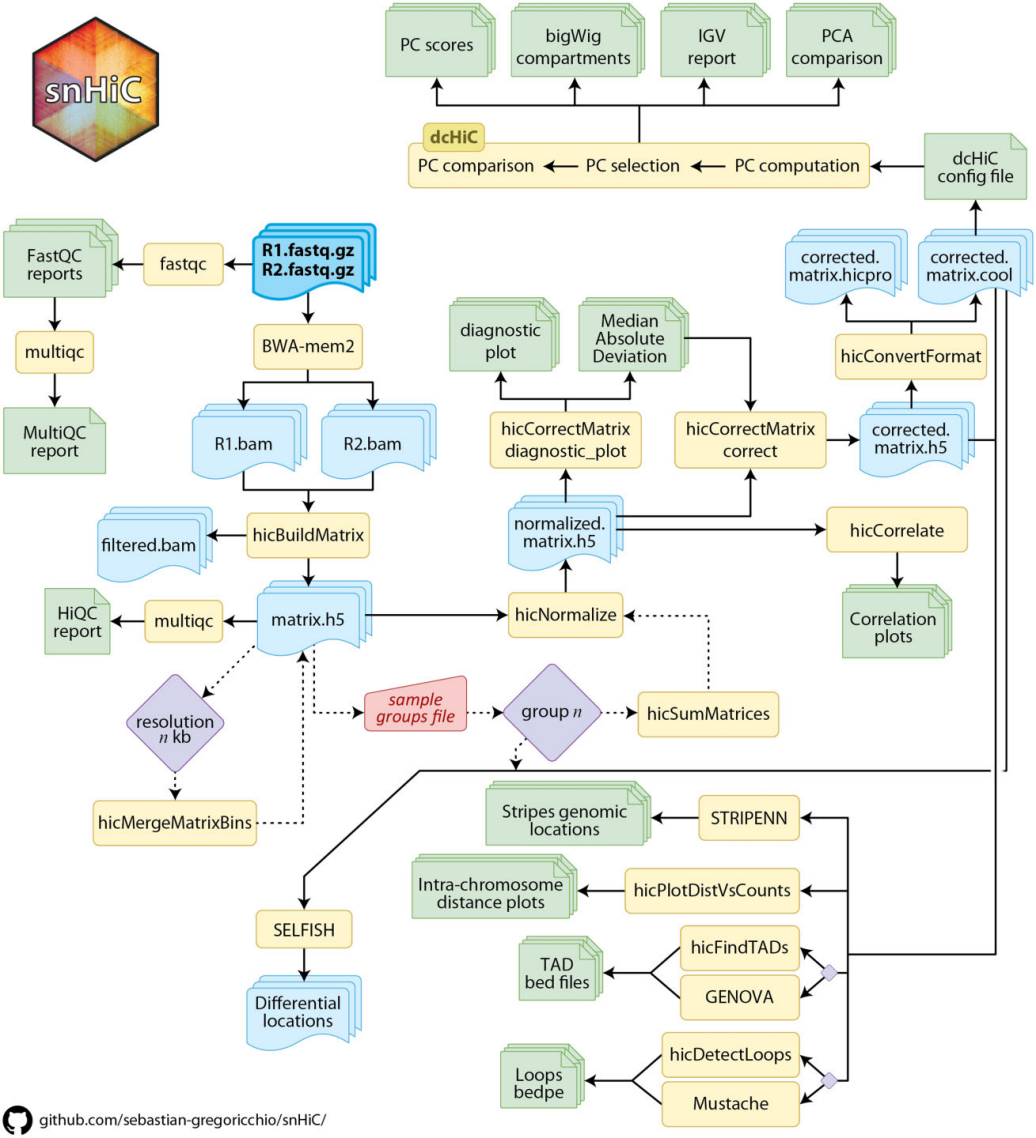
*snHiC* pipeline workflow. Sequential steps are performed in the *snHiC* pipeline starting from paired-end.fastq files (indicated by the bold box border). Software/tools/functions used in each step are indicated by rectangular boxes, while output files generated are indicated in either folded-corner boxes (text files and plots) or wave-bottom boxes (Hi-C data files, such as alignment files or contact matrices). Computational interactions are indicated by diamonds, while key user defined parameters collected from the groups_configuration file are contained in the trapezoid box

Our pipeline, *snHiC*, is a workflow organized in different sequential steps (rules) managed by a snakemake system (Fig. 1 and Supplementary Data). The input data required for the pipeline are paired-end fastq files that are mapped to the reference genome and subsequently processed to generate normalized and corrected contact matrices, to ensure reproducibility and consistency in output data (as opposed to preprocessed contact matrices). Subsequently, the contact matrices are then used to detect TADs, loops, stripes and A/B compartments. Furthermore, *snHiC* can also perform differential Hi-C chromatin interactions and compartments analyses between sample groups.

To ensure ease of use and stability, we provide a .yaml file to build a custom conda environment which is available in the *snHiC*’s GitHub repository (snHiC/workflow/envs/snHiC_conda_env_stable.yaml).

The minimal pipeline consists of a snakemake file, containing the rules to be executed, and a yaml-formatted configuration file. The latter contains all the parameters that can be customized by the user (for details, see the dedicate GitHub Wiki) and is subdivided in two parts: (i) experiment-specific parameters (e.g., reference genome, restriction enzyme used, inclusion or not of specific analyses); (ii) default parameters suitable for most of the experiments (e.g., normalization and correction methods, filters, software-specific parameters). If the user aims to perform grouped analyses, a sample configuration table is required. This table indicates to which group each sample belongs and is used to define which samples should be merged and analyzed together (e.g., replicates, tissue type, experimental conditions, etc.).

### 2.1 Fastq mapping and contact matrices

To reduce computation time, the fastq files are aligned using the accelerated version of the Burrows-Wheeler Aligner (*BWA*) (Vasimuddin *et al.*, 2019). In order to account for the chimeric reads generated by the Hi-C library preparation procedure, specific BWA options are used: ‘*bwa-mem2 mem -A1 -B4 -E50 -L0*’.

The aligned reads are then processed using *HiCExplorer* (Wolff *et al.*, 2020) to generate Hi-C contact matrices (*hicBuildMatrix*) in .h5/.cool/.hicpro format which are then normalized (*hicNormalize*) among all the samples and corrected for technical biases (*hicCorrectMatrix*).

Notably, matrices at multiple resolutions can be computed sequentially in one single run, by merging *n* contiguous bins in the lowest resolution matrix (*hicMergeMatrixBins*). When sample groups are specified, new group matrices are generated by summing the matrices of the individual samples (*hicSumMatrices*).

### 2.2 Hi-C quality controls and sample correlation

The *snHiC* pipeline generates multiple quality control files including a ‘diagnostic plot’ showing the distribution of the coverage per bin, a Hi-C MultiQC report (including library depth, *cis*-to-*trans* contacts distribution, length of the contacts, etc.), distribution of the intra-chromosomal distance and, sample correlation heatmaps and scatter plots.

Differential chromatin interactions among groups can be performed for each chromosome by *SELFISH* (Ardakany *et al.*, 2019).

### 2.3 TADs, loops, stripes and compartments calling

For each sample/group, TADs [*HiCExplorer* or *GENOVA* (van der Weide *et al.*, 2021)], loops [*HiCExplorer* or *Mustache* (Roayaei Ardakany *et al.*, 2020)] and stripes [*STRIPENN* (Yoon *et al.*, 2022)].

The A/B compartment calling and differential compartment analyses are performed by *dcHiC* (Chakraborty *et al.*, 2022) at all user-defined Hi-C resolutions. This tool allows for the selection of the best Principal Component (PC) in calling compartments, performs multivariate differential comparison of Hi-C datasets at high-resolution (up to 5 kb), and provides IGV-reports to directly visualize the results.

## 3 Discussion

In this work, we introduce *snHiC*, a new snakemake-based pipeline to facilitate Hi-C data analyses. The strength of this workflow is that automates and standardizes the processing of Hi-C data, providing an all-in-one tool to perform diverse analysis types (i.e. TADs, loops, stripes) as well as inter-group differential analyses (i.e. compartments and chromatin interactions).

Furthermore, the outputs of this pipeline are compatible with the most commonly used Hi-C visualization tools/packages—available in the conda environment—such as *GENOVA* (van der Weide *et al.*, 2021), *HiCPlotter* (Akdemir and Chin, 2015) and *pyGenomeTracks* (Lopez-Delisle *et al.*, 2021).

## Supplementary Material

vbad080_Supplementary_Data
