# Supplementary material for: snHiC: a complete and simplified snakemake pipeline for grouped Hi-C data analysis
Source: Bioinform Adv. 2023 Jun 21;3(1):vbad080. doi: 10.1093/bioadv/vbad080 (PMC10307938; doi:10.1093/bioadv/vbad080)
Supplement: vbad080_Supplementary_Data [file vbad080_supplementary_data.pdf]

snHiC: supplementary information

Supplementary material for the publication: "*snHiC*: a complete and simplified snakemake pipeline for grouped Hi-C data analysis"

Sebastian Gregoricchio[1,*], Wilbert Zwart[1,*]

[1]Division of Oncogenomics, Netherlands Cancer Institute, Oncode Institute, Plesmanlaan 121, 1065CX, Amsterdam, The Netherlands

*To whom correspondence should be addressed.

Abstract

Summary

Genome-wide chromosome conformation capture (Hi-C) is a technique that allows the study of 3D genome organization. Despite being widely used, analysis of Hi-C data is technically challenging and involves several time-consuming steps that often require manual involvement making it error prone, potentially affecting data reproducibility. In order to facilitate and simplify these analyses we implemented *snHiC*, a snakemake-based pipeline that allows for the generation of contact matrices at multiple resolutions in one single run, aggregation of individual samples into user-specified groups, detection of domains, compartments, loops and stripes, and performance of differential compartment and chromatin interaction analyses.

Availability

Source code is freely available at https://github.com/sebastian-gregoricchio/snHiC. A yaml-formatted file (snHiC/workflow/envs/snHiC_conda_env_stable.yaml) is available to build a compatible conda environment.

Contact

s.gregoricchio@nki.nl or w.zwart@nki.nl

Table of contents

1. Documentation....2
2. Feature comparison with other Hi-C pipelines 2
3. Resources and performance 2
4. Package history and releases....4
5. Contact....4
6. License 4

1. Documentation

A detailed documentation of the pipeline can be found at the dedicated GitHub Wiki.

2. Feature comparison with other Hi-C pipelines

Supplementary Table 1 | *Comparison of available features in different Hi-C analyses pipelines*

	Major outputs								
**Pipeline**	**Matrix formats**	**Multiple resolutions**	**Group analyses**	**TAD**	**Loops**	**Stripes**	**Differential contacts**	**Compartments**	**Differential Compartments**
snHiC	.h5, .cool, .hicpro	✔	✔	✔	✔	✔	✔	✔	✔
Juicer	.hic	✔		secondary step	secondary step			secondary step	
FAN-C	.hic	✔		secondary step	secondary step			secondary step	
HiC-Pro	.hicpro								

3. Resources and performance

Hereafter is reported a summary of *snHiC* performances for each rule as tested on:

- Samples: 4
- Groups: 2 (2 samples per group)
- Resolutions: 10, 20, 50, 100, 1000 kb
- Cores provided: 10
- System: HPC (GNU/Linux, x86_64), 165-Ubuntu SMP Tue Apr 18 08:53:12 UTC 2023 (5.4.0-148-generic)

Data are accessible at the *snHiC* google drive and represent a down-sampling of previously published data from San Martin *et al.* *(JCB* 2022. doi: 10.1083/jcb.202104108; GEO accession number: GSE172099):

a) DU145_rep1 (SRR14239814)
b) DU145_rep2 (SRR14239815)
c) PC3_rep1 (SRR14239816)
d) PC3_rep2 (SRR14239817)

The output of *snHiC* analyses performed on this data is downloadable at the *snHiC* google drive: individual samples and grouped analyses.

Supplementary Table 2 | *Benchmark summary*

Rule	N steps	Tot Running Time (min)	Tot Running Time (dd.hh.mm.ss)	Max physical mem (GB)	Max virtual mem (GB)	Average mean load
A_fastQC_raw	8	6.5	6M 32s	0.2	3.3	77.7
B_multiQC_raw	1	0.1	8s	0.1	0.2	33.1
C_bwa_align	8	30.9	30M 55s	21.6	37.8	442.5
D_generate_restriction_file_and_get_chrSizes	1	2.5	2M 30s	1.4	1.6	90.2
E1_interaction_matrix_and_bam_generation_at_smallest_resolution	4	64.8	1H 4M 49s	44.7	48.6	116.4
E2_multiQC_report_for_HiC_matrices	1	0.1	7s	0.1	0.3	62.2
E3_merging_interaction_matrix_bins_for_all_resolutions	16	7.8	7M 47s	0.5	0.6	56.4
F1_matrices_normalization	5	1.8	1M 47s	1.3	1.3	71.9
F2_samples_correlation	1	1.5	1M 29s	1	1	87
G1_matrices_correction__diagnosticPlot_and_MAD	20	3.3	3M 18s	0.5	0.6	67.6
G2_matrices_correction__getting_threshold_values	20	0.1	5s	0	0	0
G3_matrices_correction__correction	20	14.1	14M 7s	0.7	0.8	81.6
H1_matrices_format_conversion__cool	1	1.3	1M 15s	0.5	0.6	57.5
H2_matrices_format_conversion__hicpro	1	4.1	4M 3s	0.3	0.4	91
I_call_TADs_HiCexplorer	20	63.7	1H 3M 39s	6	6.9	84.5
J_plotting_intraChr_distances	5	5.3	5M 20s	0.8	0.8	77.3
L1_sum_matrices_by_group	1	1.8	1M 49s	1.2	10.4	71.7
L2_merging_grouped_interaction_matrix_bins_for_all_resolutions	8	4.4	4M 21s	0.6	0.6	49.9
M_grouped_matrices_normalization	5	1	1M 0s	0.8	0.8	72.4
N1_summed_matrices_correction__diagnosticPlot_and_MAD	10	1.7	1M 42s	0.5	0.6	74.3
N2_summed_matrices_correction__getting_threshold_values	10	0	0s	0	0	0
N3_summed_matrices_correction__correction	10	5.4	5M 21s	0.8	0.9	77.6
N4_summed_matrices_correction__cool_conversion	1	1	57s	0.8	0.9	75.9
N5_summed_matrices_correction__hicpro_conversion	1	3.6	3M 35s	0.3	0.4	89.4
O_call_TADs_on_summed_matrices_HiCexplorer	10	30.5	30M 27s	5.7	6.8	81.9
P_detect_loops_singleSamples_HiCexplorer	8	72.7	1H 12M 39s	3.2	5.3	3.7

Q_detect_loops_groupedSamples_HiCexplorer	4	35.9	35M 53s	3.7	5.9	3.9
R1_detect_compartments_dcHiC_singleSamples__inputFile_all_vs_all	1	0	0s	0.1	9.3	0
R2_detect_compartments_dcHiC_singleSamples__call_compartments	1	258.1	4H 18M 7s	2.1	14.7	11.9
R3_detect_compartments_dcHiC_singleSamples__bedGraphToBigWig	1	0.2	13s	0.1	0.1	0.8
R4_detect_compartments_dcHiC_singleSamples__call_compartments_combos	1	10	9M 57s	0.5	3.1	286.9
R5_detect_compartments_dcHiC_singleSamples__bedGraphToBigWig_combos	1	0.2	13s	0.1	0.1	0.6
S1_detect_compartments_dcHiC_groupedSamples__inputFile_all_vs_all	1	0	0s	0.1	9.2	0
S2_detect_compartments_dcHiC_groupedSamples__call_compartments	1	148.7	2H 28M 42s	0.9	7.1	3.9
S3_detect_compartments_dcHiC_groupedSamples__bedGraphToBigWig	1	0.1	8s	0.1	0.1	0.8
S4_detect_compartments_dcHiC_groupedSamples__call_compartments_combos	1	3.1	3M 3s	0.5	3.1	135.7
S5_detect_compartments_dcHiC_groupedSamples__bedGraphToBigWig_combos	1	0.1	8s	0.1	0.1	0.5
T_differential_contacts_SELFISH_groupedSamples	5	150	2H 29M 57s	62.7	65.5	38.9
U1_stripe_detection_STRIPPEN_singleSamples	4	430.6	7H 10M 33s	1.1	7	196.4
U2_stripe_detection_STRIPPEN_groupedSamples	2	172.8	2H 52M 49s	1.3	7	229.9
SUMMARY	***221***	***1539.8***	***1d 1H 39M 48S***	***62.7***	***65.5***	***75.1***

4. Package history and releases

A list of all releases and respective description of changes applied could be found here.

5. Contact

For any suggestion, bug fixing, commentary please report it in the issues/request tab of the *snHiC* GitHub repository.

6. License

This pipeline is under a GNU General Public License (version 3).
